# The predictive value of baseline systemic inflammation response index and systemic immune-inflammation index for the risk of infection within 6 months following initial immunosuppressive treatment in patients with ANCA-associated vasculitis

**DOI:** 10.3389/fimmu.2026.1718901

**Published:** 2026-02-09

**Authors:** Yujia Wang, Kaiqi Huang, Caiming Chen, Zigui Zheng, Kunmei Lai, Jianxin Wan, Yanfang Xu

**Affiliations:** 1 Department of Nephrology, Blood Purification Research Center, The First Affiliated Hospital, Fujian Medical University, Fuzhou, Fujian, China; 2 Research Center for Metabolic Chronic Kidney Disease, The First Affiliated Hospital, Fujian Medical University, Fuzhou, Fujian, China; 3 Department of Nephrology, National Regional Medical Center, Binhai Campus of the First Affiliated Hospital, Fujian Medical University, Fuzhou, Fujian, China

**Keywords:** ANCA-associated vasculitis, infection, predictive model, systemic immune-inflammation index (SII), systemic inflammation response index (SIRI)

## Abstract

**Background:**

Patients with ANCA-associated vasculitis (AAV) face a high risk of severe infections, particularly within the first six months of immunosuppressive therapy, contributing significantly to early mortality. The systemic immune-inflammation index (SII) and systemic inflammation response index (SIRI) are integrative biomarkers reflecting inflammatory and immune status. This study aims to develop a model based on baseline assessment and early-treatment parameters to predict infection risk in AAV patients.

**Methods:**

In this retrospective cohort study, 185 AAV patients from a single center were enrolled. Clinical and laboratory data at diagnosis, along with treatment details were collected, and the systemic immune-inflammation index (SII) and systemic inflammation response index (SIRI) were calculated. The primary endpoint was the occurrence of infection within 6 months. Univariate and multivariable logistic regression were used to identify independent risk factors. Receiver operating characteristic (ROC) curve analysis and the area under the curve (AUC) were applied to assess the predictive performance. A nomogram was developed based on the multivariable model.

**Results:**

Within 6 months, 77 patients (41.6%) developed infections, primarily respiratory (77.9%). Multivariable analysis identified five independent predictors: older age (OR = 1.040, 95% CI: 1.006-1.076, p=0.021), higher SII (OR = 1.000, 95% CI: 1.000-1.001, p=0.037), higher SIRI (OR = 1.293, 95% CI: 1.039-1.610, p=0.021), lower serum albumin (OR = 0.898, 95% CI: 0.826-0.975, p=0.011), and a higher cumulative dose of prednisone within the first month (OR = 1.002, 95% CI: 1.001-1.004, p=0.007). The combined model incorporating all five factors showed an improved area under the curve (AUC = 0.825) compared to the “clinical-only” model comprising age, albumin, and cumulative dose of prednisone within the first month (AUC = 0.741). By integrating the five independent predictors, the nomogram yields a quantitative estimate of infection risk through the summation of points assigned to each variable.

**Conclusion:**

Pre-treatment SII and SIRI are independent predictors of early infection in AAV patients. The developed nomogram, which integrates these indices with age, albumin and cumulative dose of prednisone within the first month, serves as an exploratory assessment tool for individualized risk.

## 1 Introduction

Anti-neutrophil cytoplasmic antibody (ANCA)-associated vasculitis (AAV) is a group of systemic autoimmune disorders characterized by inflammation of small blood vessels and the presence of circulating ANCAs, primarily targeting myeloperoxidase (MPO) or proteinase 3 (PR3) (1). Genetic predispositions, environmental triggers, and dysregulated immune responses further play critical roles in disease development and progression (2).

Patients with AAV are highly susceptible to infections during immunosuppressive therapy, which significantly contributes to early morbidity and mortality. A large retrospective study of 415 AAV patients revealed that 53.0% experienced at least one infection within the first three months after diagnosis, with respiratory infections being the most common (3). This high early risk is corroborated by the RAVE trial analysis, which found that 82% of severe infections occurred within the first 6 months after treatment initiation, with respiratory tract infections constituting the majority (4). Beyond their high incidence, these infections carry a lethal punch—emerging as a leading driver of first-year mortality, with infected patients dying at more than double the rate of their uninfected counterparts (3). The compromised immune state-due both to active vasculitis and intensive immunosuppression-facilitates severe bacterial, viral, and fungal infections, underscoring the critical need for proactive monitoring and risk stratification.

The systemic immune-inflammation index (SII) and systemic inflammation response index (SIRI) are integrative biomarkers derived from peripheral cell counts-including neutrophils, platelets, lymphocytes, and monocytes-that reflect systemic inflammatory and immune status. Recently, these indices have gained relevance in autoimmune diseases. For instance, elevated SII has been significantly associated with an increased risk of rheumatoid arthritis (RA), suggesting its utility in reflecting underlying inflammatory burden and immune dysregulation in autoimmune conditions (5). Similarly, SIRI has been identified as a mediator in the relationship between obesity and psoriasis, highlighting its role in linking metabolic imbalance to immune-mediated inflammation (6). In AAV, these markers may help capture the complex interplay between inflammatory activation and immune competence. Elevated SII and SIRI may indicate an imbalance in immune cells, which could potentially compromise the body’s defense mechanisms (7, 8). This dysregulation might be associated with a higher susceptibility to infections, suggesting that these indices could serve as potential markers for infection risk in autoimmune and inflammatory contexts.

Predicting infection risk before treatment initiation is critical for personalizing management. The pre-treatment levels of SII and SIRI-reflecting underlying inflammatory burden and immune dysregulation-may help stratify infection risk more accurately. Integrating these indices as well as early-treatment variables into a predictive model could identify high-risk patients who might benefit from enhanced prophylaxis, closer monitoring, or tailored immunosuppression, while minimizing unnecessary interventions in low-risk individuals. This study aims to develop such a model using relevant pre-treatment and early-treatment data, including SII and SIRI, to forecast infection within the first 6 months and improve early clinical outcomes in AAV.

## 2 Methods

### 2.1 Study population

A retrospective cohort study was conducted on patients diagnosed with AAV at the First Affiliated Hospital of Fujian Medical University between January 2014 and March 2025. The study adhered to the principles of the Declaration of Helsinki and was approved by the hospital’s Ethics Committee (protocol code: [2023]-180). Written informed consent was obtained from all participants. All patients were diagnosed based on clinical and laboratory features consistent with the definitions of AAV as per the 2012 Chapel Hill Consensus Conference Nomenclature for vasculitides, and were subsequently reclassified according to the 2022 American College of Rheumatology/European Alliance of Associations for Rheumatology (ACR/EULAR) classification criteria. Patients were excluded if they met any of the following criteria: (1) presence of a confirmed infection at initial diagnosis; (2) history of kidney transplantation; (3) comorbid autoimmune diseases; (4) absence of immunosuppressive therapy; or (5) incomplete clinical data in the medical records.

### 2.2 Data collection

Demographic and clinical data were retrospectively collected from the electronic medical record systems of our hospital. The extracted information included medical history, laboratory results at diagnosis and during follow-up, histopathological findings, and therapeutic regimens. All patients underwent standardized laboratory testing upon admission. The estimated glomerular filtration rate (eGFR) was calculated using the 2009 CKD-EPI equation. The systemic immune-inflammation index (SII) and systemic inflammation response index (SIRI) were calculated based on admission laboratory results using the following formulas: SII = (neutrophil count × platelet count)/lymphocyte count; SIRI = (neutrophil count × monocyte count)/lymphocyte count. The presence of ANCA was detected by indirect immunofluorescence, and anti-MPO along with anti-PR3 antibodies were quantified using enzyme-linked immunosorbent assay (ELISA). Treatment strategies for ANCA-associated vasculitis (AAV) at our center were primarily guided by the Kidney Disease: Improving Global Outcomes (KDIGO) guidelines and were further tailored based on individualized patient factors, such as the occurrence of infections or tolerance to glucocorticoids or immunosuppressive agents. Induction therapy generally consisted of cyclophosphamide or rituximab in combination with glucocorticoids or mycophenolate mofetil (for patients without significant organ involvement). For patients with severe organ involvement, a regimen combining cyclophosphamide and glucocorticoids, supplemented with plasma exchange, was preferred.

### 2.3 Endpoint assessment

The primary endpoint was the occurrence of infection within the first 6 months following treatment initiation. All documented infection episodes were registered based on medical record review. An infectious event was defined as either a serious infection (requiring intravenous antibiotics and/or hospitalization) or a non-serious infection (managed with oral antimicrobials in an outpatient setting). Diagnosis of infection was independently assessed by two experienced clinicians through comprehensive evaluation of clinical manifestations, laboratory results, imaging findings, serological tests, and pathogen culture results when available.

### 2.4 Statistical analysis

Continuous variables were expressed as mean ± standard deviation (SD) or median (interquartile range, IQR) and compared using Student’s t-test or the Wilcoxon rank-sum test, as appropriate. Categorical variables were summarized as frequencies and percentages and compared using the chi-square or Fisher’s exact test. For paired comparisons within the same group (e.g., baseline vs. 1-month values), the Wilcoxon signed-rank test was used. Logistic regression analysis was used to create a multivariable prediction model for the infection risk. A two-sided p-value < 0.05 was considered statistically significant.

The predictive performance of SIRI and SII was evaluated using the area under the receiver operating characteristic curve (AUC). Nomograms were developed based on multivariate analysis results. The performance was assessed using calibration curve. Model calibration was evaluated using 1000 bootstrap resamples to correct for overfitting. All statistical analyses were performed using IBM SPSS Statistics (version 26.0), GraphPad Prism (version 8.0.2), and R software (version 4.5.2).

## 3 Results

### 3.1 Study population and baseline characteristics

**Figure 1:**
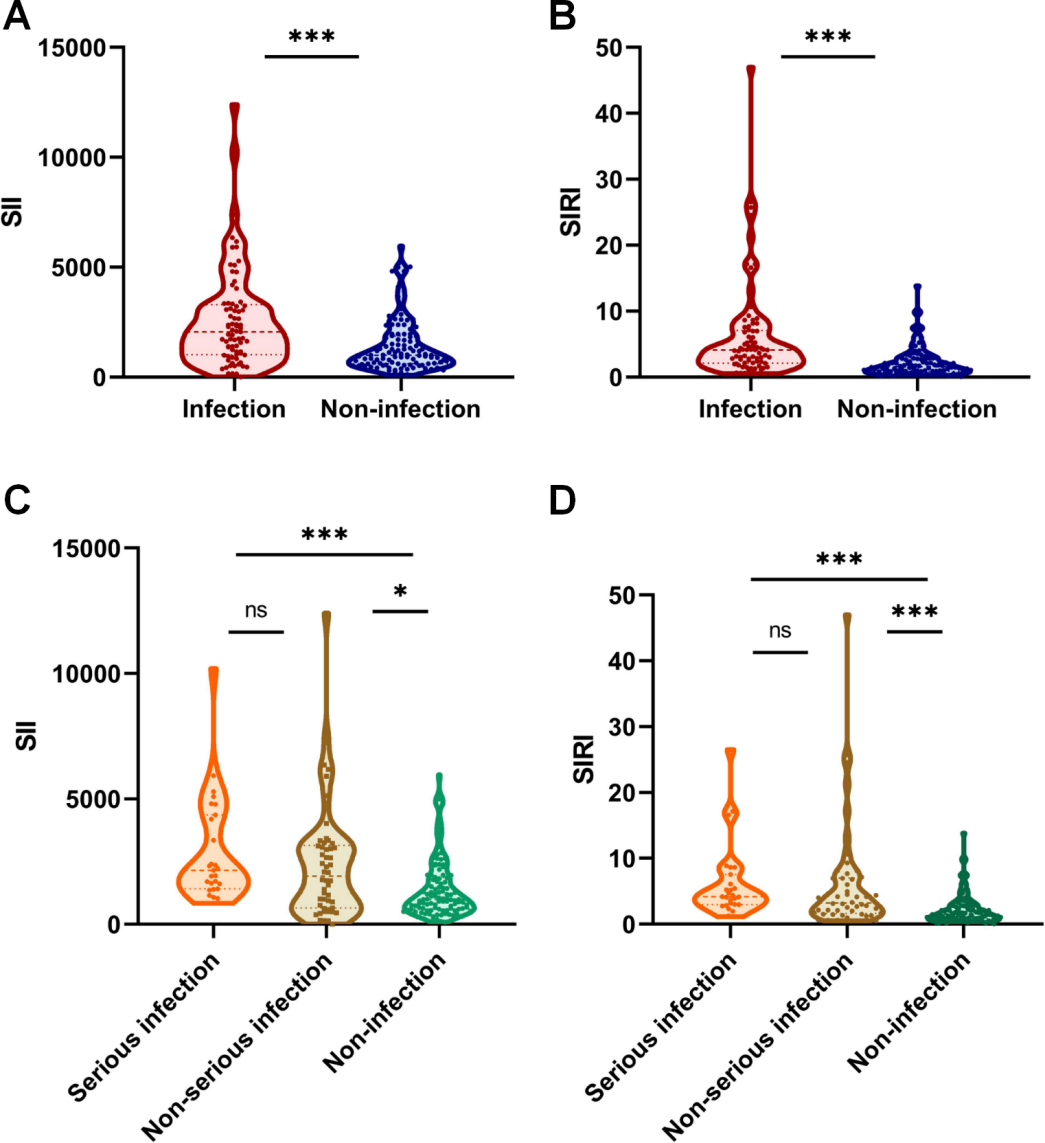
Distribution of systemic inflammatory indices at baseline. **(A, B)** Boxplots comparing baseline SII **(A)** and SIRI **(B)** levels between patients who did not develop an infection within 6 months (Non-infection, n=108) and those who did (Infection, n=77). **(C, D)** Boxplots showing SII **(C)** and SIRI **(D)** levels stratified by infection outcome: no infection (n=108), non-serious infection (n=50), and serious infection (n=27). ***p<0.001; *p<0.05; ns, not significant.

This study included a total of 185 patients with AAV who received initial immunosuppressive therapy. Within six months, 77 patients (41.6%) developed infections, with respiratory tract infections being the most common (77.9%), followed by urinary tract infections (14.3%) (**Supplementary Figure 1**). Baseline characteristics assessed at diagnosis for the overall cohort and stratified by infection status are summarized in **Table 1**. Compared to the non-infection group, patients who developed infections were significantly older (68.0 (59.0, 73.0) vs. 59.0 (48.3, 67.0) years; p< 0.001) and presented with markedly elevated systemic inflammation markers, including SII (2059.32 (1024.01, 3288.12) vs. 994.28 (607.78, 1970.85); p < 0.001) and SIRI (4.10 (2.11, 7.07) vs. 1.58 (0.87, 2.85); p< 0.001) (**Figures 1A, B**). Furthermore, the infection group demonstrated poorer renal function at baseline, reflected by lower eGFR (10.30 (5.84, 30.64) vs. 16.89 (7.27, 54.69) mL/min/1.73 m²; p=0.014), and reduced serum albumin levels (29.3 (26.6, 32.9) vs. 33.1 (29.5, 38.2) g/L; p< 0.001). Significant intergroup differences were also observed in several other baseline laboratory parameters, including serum sodium, chloride, calcium, total cholesterol, D-dimer, and CRP (all p< 0.05). In contrast, no statistically significant differences were found in sex distribution, prevalence of hypertension or diabetes mellitus, or baseline levels of total bilirubin, ALT, AST, serum globulin, serum potassium or triglycerides.

**Table 1:** Baseline characteristics in overall, infected and non-infected patients with AAV.

Variables	Total (n=185)	Infection (n=77)	Non-Infection (n=108)	P value
Age, years (median (IQR))	62.0 (62.5, 70.0)	68.0 (59.0, 73.0)	59.0 (48.3, 67.0)	**<0.001**
Male, n (%)	101 (55)	47 (61.0)	54 (50)	0.137
Hypertension, n (%)	32 (17.3)	11 (14.3)	21 (19.4)	0.346
Diabetes mellitus, n (%)	109 (58.9)	42 (54.5)	67 (62.0)	0.307
SII (median (IQR))	1292.39 (661.41, 2409.56)	2059.32 (1024.01, 3288.12)	994.28 (607.78, 1970.85)	**<0.001**
SIRI (median (IQR))	2.19 (1.15, 4.39)	4.10 (2.11, 7.07)	1.58 (0.87, 2.85)	**<0.001**
eGFR, mL/min/1.73 m^2^ (median (IQR))	13.14 (6.43, 42.68)	10.30 (5.84, 30.64)	16.89 (7.27, 54.69)	**0.014**
Total bilirubin, μmol/L (median (IQR))	4.95 (3.78, 7.40)	4.8 (3.8, 8.2)	5.0 (3.7, 6.7)	0.765
ALT, U/L(median (IQR))	12.0 (8.0, 19.25)	13.0 (8.0, 21.0)	12.0 (8.0, 19.0)	0.331
AST, U/L(median (IQR))	17.0 (13.0, 23.25)	18.0 (13.5, 29.0)	16.0 (13.0, 21.0)	0.108
Serum albumin, g/L, (median (IQR))	32.1 (28.0, 36.33)	29.3 (26.6, 32.9)	33.1 (29.5, 38.2)	**<0.001**
Serum globulin, g/L, (mean ± SD)	28.02 ± 6.66	28.85 ± 6.93	27.45 ± 6.44	0.187
K^+^, mmol/L (mean ± SD)	4.30 ± 0.65	4.36 ± 0.69	4.26 ± 0.63	0.282
Na^+^, mmol/L (median (IQR))	139.3 (135.7, 141.8)	138.4 (134.3, 141.3)	139.9 (137.1, 142.7)	**0.012**
Cl^-^, mmol/L (mean ± SD)	102.33 ± 5.96	100.87 ± 6.11	103.35 ± 5.66	**0.005**
Ca^2+^, mmol/L (median (IQR))	2.07 (1.95, 2.19)	2.03 (1.91, 2.13)	2.11 (2.00, 2.23)	**0.001**
Total cholesterol, mmol/L (median (IQR))	3.95 (3.29, 4.92)	3.70 (3.18, 4.56)	4.14 (3.36, 5.39)	**0.040**
Triglycerides, mmol/L (median (IQR))	1.35 (0.98, 1,73)	1.19 (0.91, 1.62)	1.42 (1.03, 1.77)	0.061
D-Dimer, μg/mL (median (IQR))	1.87 (0.72, 3.87)	2.90 (1.63, 5.84)	1.16 (0.47, 2.26)	**<0.001**
CRP, mg/L (median (IQR))	14.44 (4.59, 52.15)	32.33 (10.92, 66.08)	7.69 (1.56, 34.78)	**<0.001**
Treatment (1st Month)				
Corticosteroid, n (%)	163 (88.1)	72 (93.5)	91 (84.2)	0.055
Cumulative dose of prednisone, mg, (median (IQR))	805 (700, 980)	805 (770, 980)	805 (630, 980)	**0.045**
Intravenous CTX, n (%)	118 (63.7)	54 (70.1)	64 (59.2)	0.129
Cumulative dose of CTX, g, (median (IQR))	0.4 (0.0, 1.0)	0.5 (0.0, 1.0)	0.4 (0.0, 1.25)	0.675
Rituximab, n (%)	34 (18.3)	11 (14.2)	23 (21.2)	0.313
Plasma exchange	53 (28.6)	24 (31.1)	29 (26.8)	0.522

Bold values denote statistical significance.

We further stratified infected patients by severity into serious (n=27) and non-serious (n=50) infection groups. The median (IQR) SII levels were 2147.28 (1420.96, 4358.60) in the serious infection group, 1916.10 (648.71, 3148.25) in the non-serious infection group, and 994.28 (607.78, 1870.85) in the non-infection group. The median (IQR) SIRI levels were 4.20 (2.96, 7.58), 3.22 (1.65, 7.03), and 1.58 (0.87, 2.85) in the three groups, respectively (**Figures 1C, D**). Both SII and SIRI levels were significantly elevated in the serious and non-serious infection groups compared to the non-infection group. However, no statistically significant difference was observed in SII or SIRI levels between the serious and non-serious infection groups.

Treatment-related factors during the first month were also compared between groups. The use of corticosteroids showed a trend toward being more frequent in the infection group (93.5%) compared to the non-infection group (84.2%), with a p-value of 0.055. The cumulative dose of prednisone was greater in those who developed infections compared to those who did not. No significant differences were observed in the use of intravenous cyclophosphamide, cumulative cyclophosphamide dose, rituximab, or plasma exchange.

### 3.2 Changes in SII and SIRI during early treatment and their association with infection risk

To assess whether early changes in inflammatory indices predict later infection, we analyzed SII and SIRI dynamics one month after treatment initiation in 139 patients with available data at month 1 (56 with subsequent infection, 83 without). As shown in **Supplementary Table 1**, SIRI decreased significantly from baseline in the infection group (p=0.001), whereas no significant change was observed in the non-infection group (p=0.054). The percent change in SIRI (ΔSIRI%) differed significantly between the infection and non-infection groups (p=0.001). In contrast, SII did not change significantly in either group. However, in univariate logistic regression (**Supplementary Table 2**), ΔSIRI% was not significantly associated with infection risk (OR = 0.880, 95% CI: 0.733-1.056, p=0.168).

### 3.3 Univariate and multivariable logistic regression analysis for infection risk in AAV patients

To identify independent risk factors for infection, we performed logistic regression analyses. The univariate analysis (**Table 2**) revealed that age, SII, SIRI, serum albumin, sodium, chloride, D-dimer, CRP and a higher cumulative dose of prednisone within the first month were significantly associated with an increased risk of infection (p<0.05). Renal function, as reflected by eGFR, was also associated with an increased risk, approaching statistical significance (p=0.062). Given its established clinical relevance in infection risk, eGFR was retained for inclusion in the subsequent multivariable analysis alongside the statistically significant variables. These significant variables were subsequently incorporated into a multivariable logistic regression model (**Table 3**). After adjustment, five factors remained independent predictors of infection: older age (OR = 1.040, 95% CI: 1.006-1.076, p=0.021), higher SII (OR = 1.000, 95% CI: 1.000-1.001, p=0.037), higher SIRI (OR = 1.293, 95% CI: 1.039-1.610, p=0.021), lower serum albumin (OR = 0.898, 95% CI: 0.826-0.975, p=0.011), and a higher cumulative dose of prednisone (OR = 1.002, 95% CI: 1.001-1.004, p=0.007). Notably, eGFR was no longer statistically significant in the multivariable model (p=0.288).

**Table 2:** Univariate logistic regression models for infectious events in AAV patients.

Variables	OR (95%CI)	P value
Age, years	1.046 (1.021-1.071)	**<0.001**
Sex (male)	0.638 (0.353-1.155)	0.138
Hypertension	0.683 (0.308-1.514)	0.348
Diabetes mellitus	0.734 (0.406-1.329)	0.308
SII	1.000 (1.000-1.001)	**<0.001**
SIRI	1.340 (1.173-1.530)	**<0.001**
eGFR, mL/min/1.73 m^2^	0.992 (0.983-1.000)	0.062
Total bilirubin, μmol/L	0.994 (0.913-1.081)	0.884
ALT, U/L	1.010 (0.990-1.031)	0.326
AST, U/L	1.023 (1.000-1.047)	0.055
Serum albumin, g/L	0.878 (0.8270-0.932)	**<0.001**
Serum globulin, g/L	1.0332 (0.985-1.081)	0.181
K^+^, mmol/L	1.280 (0.817-2.004)	0.281
Na^+^, mmol/L	0.925 (0.869-0.985)	**0.015**
Cl^-^, mmol/L	0.930 (0.882-0.980)	**0.007**
Ca^2+^, mmol/L	1.003 (0.991-1.015)	0.660
Total cholesterol, mmol/L	0.828 (0.668-1.026)	0.085
Triglycerides, mmol/L	0.824 (0.597-1.139)	0.242
D-Dimer, μg/mL	1.107 (1.019-1.202)	**0.016**
CRP, mg/L	1.002 (1.000-1.003)	**0.045**
Corticosteroid	2.690 (0.947-7.641)	0.063
Cumulative dose of prednisone	1.001 (1.000-1.002)	**0.006**
Intravenous CTX	1.698 (0.924-3.110)	0.088
Cumulative dose of CTX	0.829 (0.472-1.460)	0.516
Rituximab	0.636 (0.291-1.390)	0.257
Plasma exchange	1.234 (0.648-2.347)	0.522

Bold values denote statistical significance.

**Table 3:** Multivariable logistic regression models for infectious events in AAV patients.

Variables	OR (95%CI)	P value
Age, years	1.040 (1.006-1.076)	**0.021**
SII	1.000 (1.000-1.001)	**0.037**
SIRI	1.293 (1.039-1.610)	**0.021**
eGFR-EPI, mL/min/1.73 m^2^	0.991 (0.975-1.007)	0.288
Serum albumin, g/L	0.898 (0.826-0.975)	**0.011**
Na^+^, mmol/L	0.973 (0.865-1.095)	0.653
Cl^-^, mmol/L	0.995 (0.899-1.100)	0.917
D-Dimer, μg/mL	0.993 (0.901-1.094)	0.886
CRP, mg/L	0.998 (0.989-1.006)	0.555
Cumulative dose of prednisone	1.002 (1.001-1.004)	**0.007**

Bold values denote statistical significance.

### 3.4 Predictive performance of inflammatory indices in AAV patients

**Figure 2:**
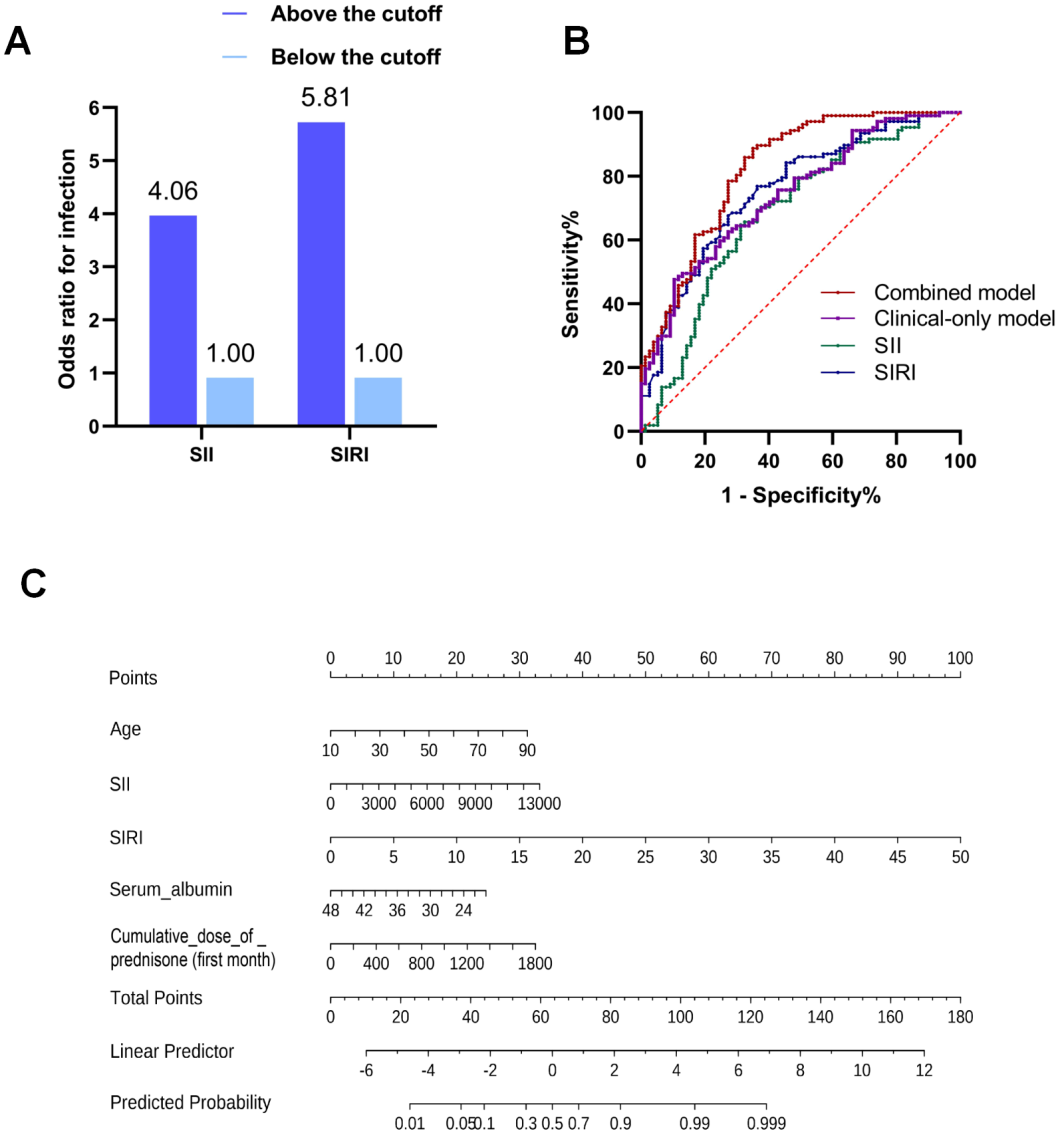
Predictive performance and clinical application of the model for infection risk within 6 months after initial immunosuppressive therapy in AAV patients. **(A)** Odds ratios for infection based on SII and SIRI groups stratified by optimal cutoff values determined by Youden index. **(B)** Receiver operating characteristic (ROC) curves evaluating the predictive accuracy of the SII, SIRI, “clinical-only” model and combined “clinical+inflammatory indices” model. **(C)** A nomogram developed to estimate the individual probability of infection.

Stratification based on optimal cutoff values determined by Youden index revealed that patients in the high-level groups for SII and SIRI faced a substantially higher risk of infection, with odds ratios of 4.06 (2.18-7.58) (p< 0.001)and 5.81 (3.04-11.07) (p< 0.001), respectively, compared to the low-level reference groups (**Figure 2A**). Further stratification by infection severity showed that the high-level groups of SII and SIRI were associated with an increased risk of both non-serious and serious infections. For SII, the odds ratios were 2.76 (1.39-5.51) for non-serious infection and 10.59 (3.41-32.88) for serious infection. Corresponding odds ratios for SIRI were 4.23 (2.07-8.64) and 11.62 (4.23-31.95), respectively (**Supplementary Figure 2**). The predictive performance of these indices was further quantified by ROC analysis (**Figure 2B**). The AUC for SII was 0.684, and for SIRI, it was 0.756, demonstrating their moderate predictive ability for infection. To assess the added value of SII/SIRI over standard clinical parameters, we constructed and compared two models: a “clinical-only” model comprising age, albumin, and cumulative dose of prednisone within the first month (AUC = 0.741), and a combined “clinical+inflammatory indices” model incorporating SII, SIRI, age, albumin, and cumulative dose of prednisone within the first month. Notably, the combined model showed improved discriminative ability in this cohort, with an AUC of 0.825. Based on this combined model, we constructed an exploratory nomogram to visually represent the contribution of each variable to the estimated individual probability of infection risk within 6 months (**Figure 2C**). The nomogram integrates the five independent predictors, allowing for a quantitative assessment of infection risk by summing the points assigned to each variable. The calibration curve suggested a reasonable agreement between the predicted and actual probabilities (**Supplementary Figure 3**).

### 3.5 Correlation of SII and SIRI with disease activity markers

To investigate the relationship between inflammatory indices and disease severity, we analyzed the correlations of pre-treatment SII and SIRI with key disease activity markers (**Supplementary Figure 4**). SII was positively correlated with CRP (r=0.432, p<0.001) and ESR (r=0.315, p=0.004). In contrast, no significant correlation was observed between SII and BVAS (r=-0.066, p=0.452) or baseline serum creatinine (r=-0.115, p=0.116). Regarding SIRI, a positive correlation was also found with CRP (r=0.407, p<0.001). Although a trend toward a positive association with BVAS was noted (r=0.162, p=0.062), it did not reach statistical significance. SIRI was not significantly correlated with ESR (r=0.053, p=0.639) or baseline serum creatinine (r=0.038, p=0.604).

## 4 Discussion

In the present study, we observed that approximately 41.6% of patients with AAV developed infections within the first 6 months following the initiation of immunosuppressive therapy. Respiratory tract infections were the most frequently encountered type. These findings are consistent with previous reports in the literature (3, 4, 9). The high incidence of early infections, particularly respiratory infections, in AAV patients can be attributed to several interrelated factors. Firstly, AAV intrinsically and frequently involves the lungs (10), which can compromise local architectural integrity and defense mechanisms. A novel murine model demonstrated that MPO-ANCA IgG alone can initiate a rapid cascade of pulmonary capillaritis and microabscess formation within days, which evolves into granulomatous lesions, mirroring the pathology seen in human GPA (11). This ANCA-triggered neutrophilic inflammation directly disrupts the alveolar-capillary barrier, creating portals for pathogen entry and establishing a pro-inflammatory microenvironment conducive to secondary infection. Secondly, the intensive immunosuppressive regimens, including high-dose glucocorticoids and agents such as cyclophosphamide or rituximab, significantly impair both cellular and humoral immunity, increasing susceptibility to opportunistic pathogens. The convergence of this iatrogenic immunosuppression upon a lung already rendered vulnerable by active ANCA-mediated inflammation creates a perfect storm for respiratory infections.

Our analysis suggested that elevated levels of both SII and SIRI at diagnosis are associated with an increased risk of developing infections within the initial 6-month period following immunosuppressive therapy. The SII and SIRI are composite hematological indices that reflect the systemic inflammatory burden and immune dysregulation. In the context of AAV, recent evidence has demonstrated that these indices are significantly elevated compared to healthy controls and correlate positively with established disease activity scores such as the Birmingham Vasculitis Activity Score (BVAS) and the Five-Factor Score (FFS) (12). Notably, SIRI exhibited the highest discriminatory power for distinguishing AAV patients from controls, underscoring its sensitivity to the profound neutrophilic and monocytic activation characteristic of AAV pathogenesis. Consistent with their role in reflecting systemic inflammation, both SII and SIRI in our cohort showed strong positive correlations with the acute-phase reactant CRP, and SII also correlated with ESR. However, their correlations with the composite clinical activity score BVAS were weaker; SIRI showed a positive trend that did not reach statistical significance, while SII did not show a significant correlation. This pattern suggests that while SII and SIRI are sensitive markers of the concomitant systemic inflammatory burden, their association with the multifaceted clinical disease activity captured by BVAS may be more variable or context-dependent in different patient populations.

Our study identified the cumulative prednisone dose as an independent predictor of infection, reinforcing the established link between glucocorticoid exposure and infection risk in AAV, as also reported in other cohorts (3). This finding underscores the importance of strategies aimed at minimizing steroid burden. Our findings regarding SII and SIRI as predictors of infection risk also prompt consideration of their potential utility in guiding therapeutic strategies aimed at minimizing glucocorticoid exposure, such as the use of avacopan. Avacopan, a complement C5a receptor inhibitor, has been demonstrated to facilitate a substantial reduction in cumulative glucocorticoid dose compared with a standard prednisone taper (the ADVOCATE trial) and to ameliorate steroid-related toxicity across multiple domains (13). Notably, in that trial, while the Cumulative Worsening Score in the infection domain at 26 weeks was numerically lower in the avacopan group than in the prednisone group, this difference did not reach statistical significance, though it suggests a potential trend toward reduced infection-related toxicity with avacopan (13). Real-world data further confirm its steroid-sparing effect, showing significantly lower cumulative prednisone doses at 6 and 12 months in avacopan-treated patients (14). However, neither the pivotal trial nor real-world studies have demonstrated a statistically significant reduction in overall infection rates with avacopan compared to conventional steroid-based regimens over similar follow-up periods (13, 14). This indicates that while reducing the glucocorticoid burden is intrinsically beneficial and may mitigate certain steroid toxicities-including potential infection risk-the complex etiology of infections in AAV, involving factors such as disease activity, underlying immune dysregulation, and iatrogenic immunosuppression, may not be fully addressed by steroid reduction alone in the short term. In this context, our predictive model-incorporating SII, SIRI, age, albumin and cumulative dose of prednisone-may serve as a supplementary tool to identify patients at elevated infection risk. By highlighting individuals with heightened inflammatory burden alongside clinical vulnerabilities, it could inform a more individualized management approach. For such high-risk patients, earlier consideration of steroid-sparing agents like avacopan may help reduce one modifiable risk factor (cumulative glucocorticoid exposure), while also prompting closer infection monitoring.

Further analysis stratified by infection severity offered additional insights. While median SII and SIRI levels were not significantly different between serious and non-serious infection groups, risk stratification using optimal cutoffs revealed a gradient. The odds ratios for serious infection in the high-level groups were notably higher than those for non-serious infection. This observation is supported by findings in other inflammatory conditions where elevated SII and SIRI have been shown to predict critical outcomes such as ICU admission (15). This may suggest that exceeding a specific inflammatory threshold identifies patients at disproportionately higher risk for severe complications, although the clinical severity of an established infection is likely influenced by multiple subsequent factors. Future large-scale studies are needed to establish a robust threshold for these indices to effectively stratify patients according to their risk of infection severity.

The findings from other studies further substantiate the role of systemic inflammatory indices as biomarkers of immune dysregulation and infection risk. In a cross-sectional analysis of NHANES 2001–2018, elevated SII was positively associated with chronic bronchitis, a condition characterized by persistent airway inflammation and immune activation (16). In a study of premature infants, elevated levels of SIRI at birth were identified as independent predictors for both neonatal respiratory distress syndrome and subsequent secondary infections, including pneumonia and sepsis (17). Similarly, in a markedly different clinical context involving adults with ST-elevation myocardial infarction (STEMI) undergoing percutaneous coronary intervention (PCI), a high SIRI at admission was a powerful independent predictor for the development of pneumonia within two weeks post-procedure (18). These findings across the age spectrum—from premature infants to elderly cardiac patients-underscore that SIRI and SII are robust biomarkers of a dysregulated immune-inflammatory state that transcends specific diseases. In AAV patients initiating immunosuppression, this pre-existing, quantifiable inflammatory burden (high SII/SIRI) likely indicates an immune system that is simultaneously overactive against self-antigens yet fundamentally compromised in its ability to mount an effective and controlled defense against pathogens. The introduction of potent immunosuppressive agents in this context may precipitously tip the balance from a hyperinflammatory state toward profound immunosuppression, thereby unmasking the latent infection risk captured by these readily available hematological indices. However, this perspective stands in contrast to the significant inverse association between SIRI and LTBI incidence reported by Wang L et al (19), highlighting a complex and potentially context-dependent relationship. The apparent contradiction may stem from key differences in study populations, such as variations in genetic background, prevalence of comorbidities, or the influence of unmeasured environmental factors that differentially shape the host immune response to M. tuberculosis.

Interestingly, we observed that SIRI decreased significantly one month after treatment initiation specifically in patients who later developed infections, whereas no such decline occurred in those without subsequent infection. This dynamic may indicate a particularly strong myelosuppressive effect of the therapy in these patients, leading to a sharp decline in innate immune effector cells. While a decrease in inflammatory indices like SIRI is generally associated with a favorable treatment response in other inflammatory conditions, such as pancreatic cancer where a declining SIRI correlates with improved outcomes after chemotherapy (20), our contrasting finding in AAV suggests a different implication. In AAV, a sharp early decline in SIRI may indicate an overshoot of immunosuppression, depleting neutrophils and monocytes crucial for frontline defense. This erosion of innate immunity could heighten vulnerability to pathogens, explaining the increased infection risk in this subgroup.

Our study also identified that advanced age, hypoalbuminemia, and a lower estimated glomerular filtration rate (eGFR) at admission were significant independent risk factors for serious infection within six months of initial immunosuppressive therapy. These findings align consistently with the established literature on infection risk in patients with AAV (9) and other diseases (21, 22). Advanced age is a well-recognized marker of immunosenescence and reduced physiological reserve. Hypoalbuminemia reflects not only poor nutritional status but also a systemic inflammatory state, which can impair immune cell function and tissue repair. Similarly, impaired renal function (low eGFR) is associated with immune dysfunction and altered pharmacokinetics of immunosuppressive drugs, potentially leading to higher cumulative exposure and toxicity.

Several limitations of our study should be acknowledged. First, this was a single-center retrospective study with a relatively modest sample size, which may restrict the generalizability of our findings and introduce selection bias. Most importantly, external validation in an independent, ideally prospective multicenter cohort is required to confirm the nomogram’s accuracy and clinical applicability before routine use. Second, although we included key clinical variables, unmeasured confounders such as nutritional status, detailed microbiological data, or prior vaccination history could influence infection risk and were not accounted for. Third, SII and SIRI reflect immune dysregulation inherent to active AAV-the same state that elevates infection risk and is targeted by immunosuppression. This overlap challenges the isolation of their independent predictive value from overall disease severity.

In conclusion, our study demonstrates that elevated pre-treatment SII and SIRI are readily accessible and independent predictors of infection within the first 6 months following initial immunosuppressive therapy in AAV patients. By integrating these novel inflammatory indices with traditional risk factors-age, serum albumin and the cumulative prednisone dose-we developed an exploratory predictive nomogram based on a single-center retrospective cohort. This model provides a preliminary, quantitative estimate of individual infection risk at diagnosis and requires further validation. Such an approach could aid in risk stratification and inform future studies on personalized monitoring strategies in this vulnerable population.

## Data Availability

The raw data supporting the conclusions of this article will be made available by the authors, without undue reservation.
