# Supplementary material for: The predictive value of baseline systemic inflammation response index and systemic immune-inflammation index for the risk of infection within 6 months following initial immunosuppressive treatment in patients with ANCA-associated vasculitis
Source: Front Immunol. 2026 Feb 9;17:1718901. doi: 10.3389/fimmu.2026.1718901 (PMC12926124; doi:10.3389/fimmu.2026.1718901)
Supplement: Supplementary file 1 [file DataSheet1.pdf]

Supplementary Material

1 Supplementary Figures

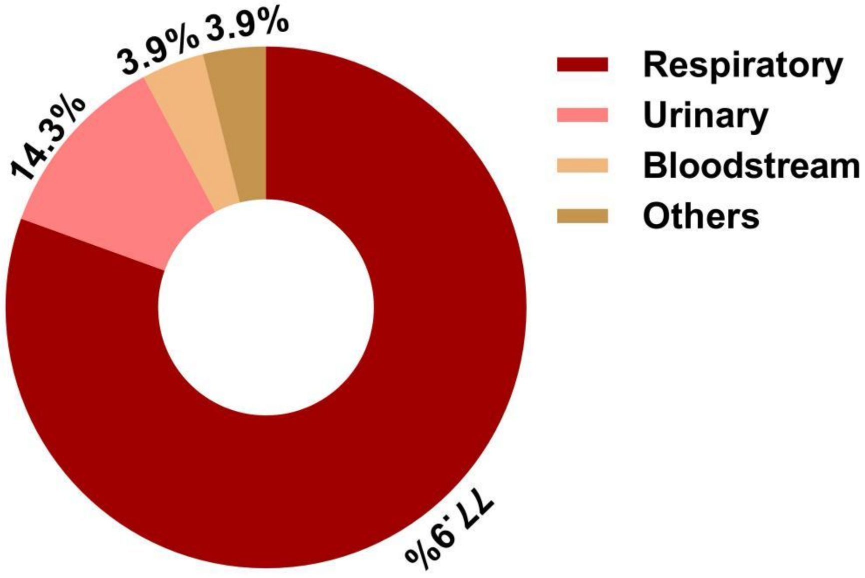

Supplemental Figure 1. Percentage of infection sites in patients with AAV

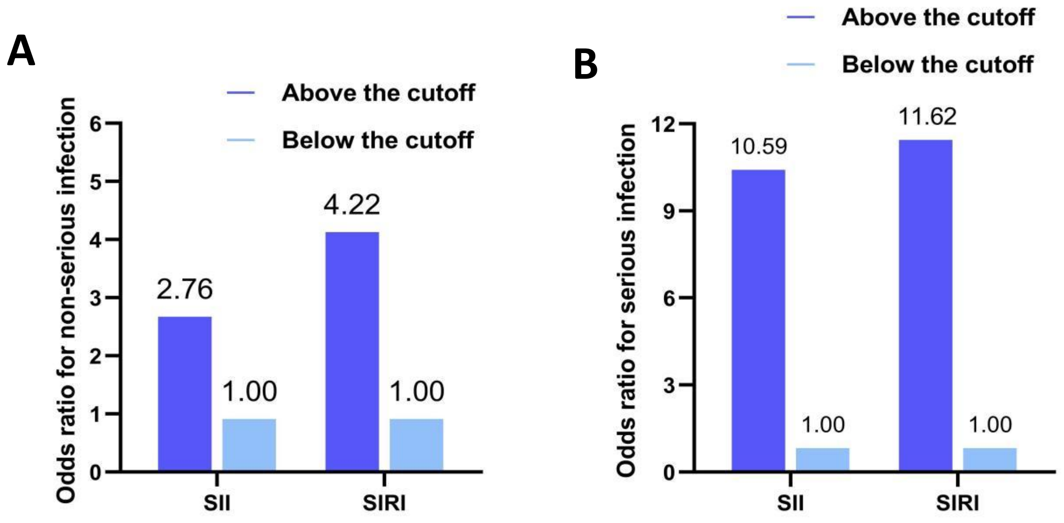

Supplemental Figure 2. Association of high SII and SIRI levels with infection severity using optimal cutoffs.

Odds ratios for non-serious(A) and serious infection(B) based on SII and SIRI groups stratified by the optimal cutoff value determined by Youden index.

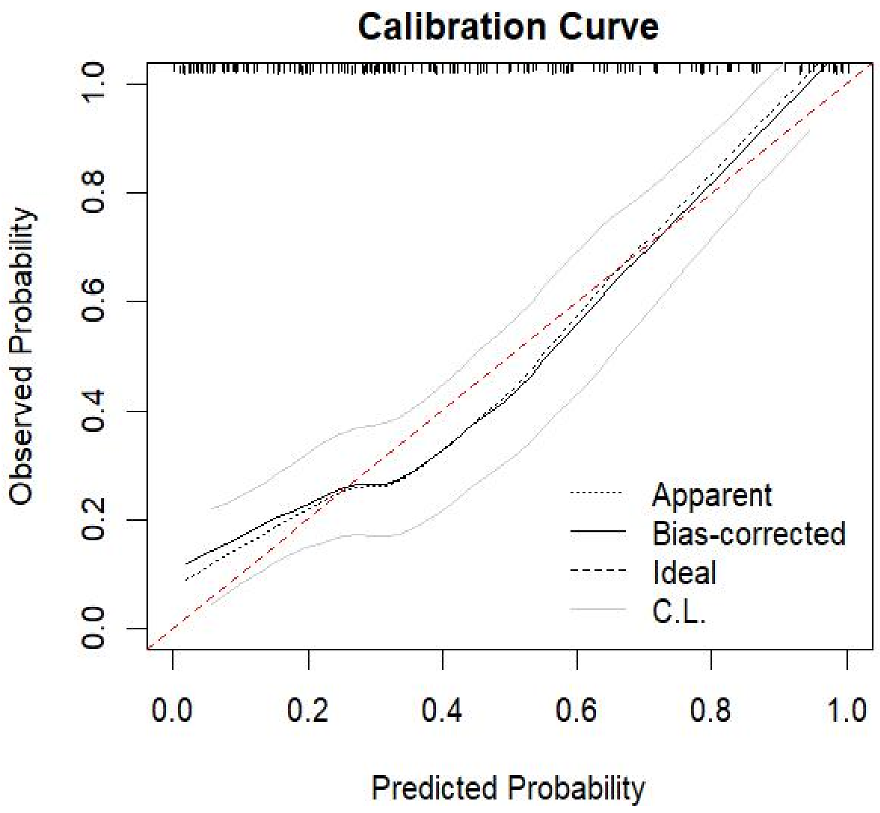

Supplemental Figure 3. Calibration curves of the nomogram model.

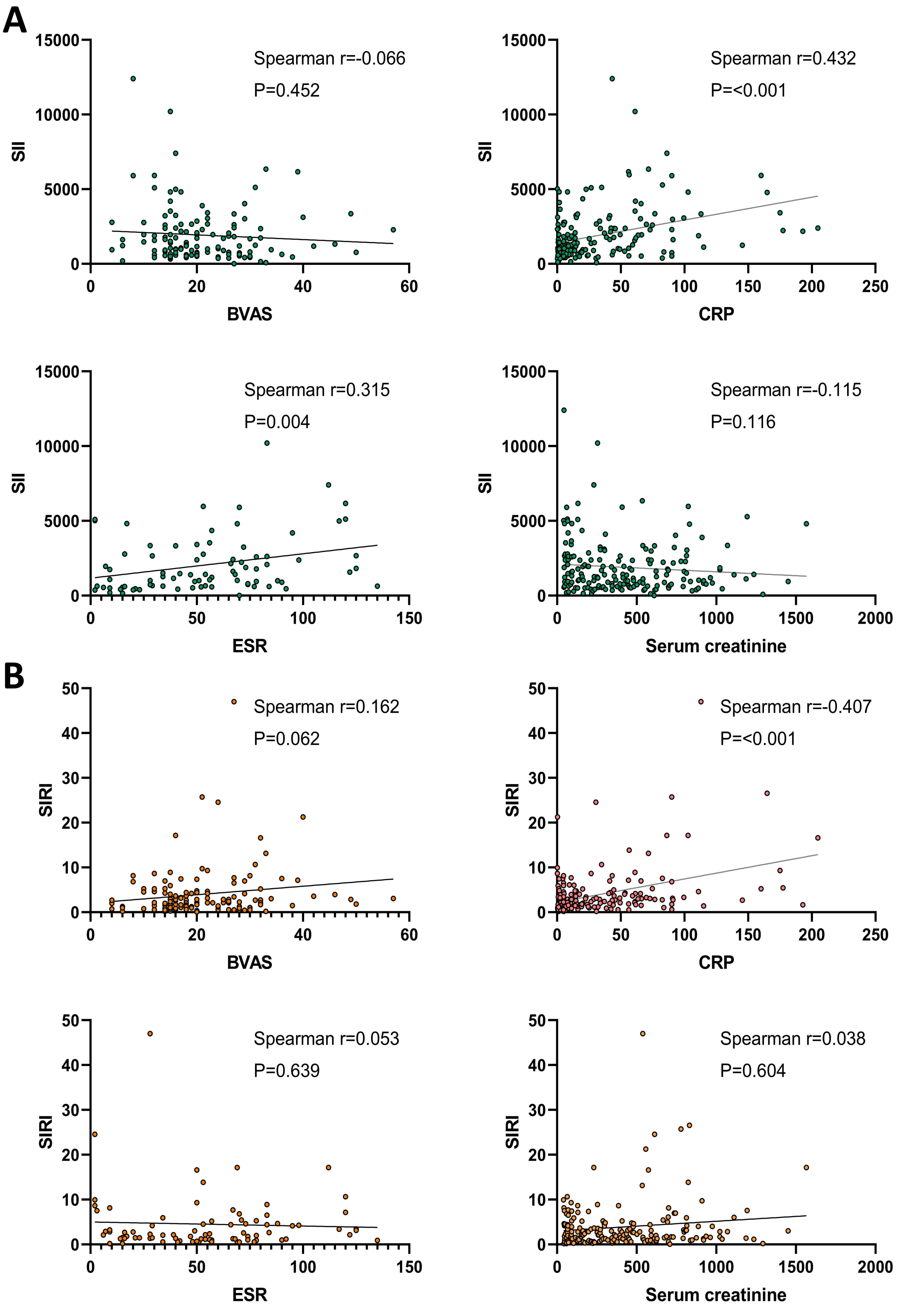

Supplemental Figure 4. The correlations between baselie SII (A) or SIRI (B) values and markers of disease severity, including BVAS, CRP, ESR, and serum creatinine.

2 Supplementary Tables

Supplementary Table 1. Changes in SII and SIRI at 1 month after treatment initiation in infected and non-infected AAV patients.

[a]P value for comparison of percentage changes between groups.

	Infection (n=56)			P	Non-Infection (n=83)			P	P[a]
Month	0	1	∆ %		0	1	∆ %		
SII	1757.99 (915.40,3008.48)	1347.14 (570.11, 2272.36)	-12.56(-73.99,70.62)	0.577	938.83 (541.62, 1504.29)	968.00 (582.77, 1947.55)	3.58(-30.28,55.44)	0.142	-
SIRI	3.79(1.64, 6.08)	2.57(1.45, 4.60)	-20.02 (-65.58,35.01)	0.001	1.56(0.90, 2.80)	2.08(0.97,3.28)	25.64(-11.69, 115.79)	0.054	0.001

Supplementary Table 2. Univariate logistic regression analysis of ∆ SIRI% for infectious events in AAV patients.

	OR (95%CI)	P value
∆ SIRI %	0.880(0.733, 1.056)	0.168
